# Deep brain stimulation and spinal cord stimulation for chronic disorders of consciousness: a decade of clinical evidence, mechanistic insights, and critical appraisal

**DOI:** 10.3389/fnhum.2026.1982589

**Published:** 2026-10-07

**Authors:** Dongsheng Liu, Kai Yang, Jianlei An, Sipeng Zhu, Yitong Jia, Yichen Zhou, Yong Wang, Weihua Cheng, Kaihan Jiang, Yi Yang, Shaoya Yin, Lei Liu

**Affiliations:** 1 Department of Neurosurgery, Aviation General Hospital, Beijing, China; 2 Department of Neurosurgery, Beijing Tiantan Hospital, Beijing, China; 3 Department of Rehabilitation Medicine, Zhujiang Hospital, Southern Medical University, Guangzhou, China; 4 Clinical College of Neurology, Neurosurgery and Neurorehabilitation, Tianjin Medical University, Tianjin, China; 5 Tianjin Key Laboratory of Cerebral Vascular and Neurodegenerative Diseases, Tianjin Neurosurgical Institute, Tianjin Medical University, Tianjin, China; 6 Department of Neurosurgery, Tianjin Huanhu Hospital, Tianjin, China

**Keywords:** central thalamus, deep brain stimulation, disorders of consciousness, mesocircuit hypothesis, minimally conscious state, neuromodulation, spinal cord stimulation, vegetative state

## Abstract

Deep brain stimulation (DBS) and spinal cord stimulation (SCS) have been investigated for over four decades as neuromodulatory treatments for chronic disorders of consciousness (DOC), including the vegetative state/unresponsive wakefulness syndrome (VS/UWS) and the minimally conscious state (MCS), for which no disease-modifying pharmacological therapy exists. This narrative review synthesizes literature published between 2016 and 2026 on the neurobiological rationale, clinical evidence, neurophysiological biomarkers, and safety of DBS and SCS for chronic DOC. The ‘mesocircuit hypothesis’, supported by human intraoperative electrophysiology and nonhuman primate causal manipulation, provides a coherent rationale for central thalamic and centromedian-parafascicular targeting. Across case series, cohort studies, and individual patient data meta-analyses, both DBS and SCS produce clinically meaningful improvement in a minority of patients, consistently more often in MCS (63–74% response) than in VS/UWS (26–30% response), with younger age and shorter injury-to-treatment interval predicting better outcomes. However, the two largest individual patient data meta-analyses of DBS concur that current evidence is insufficient to conclude that stimulation alters the natural history of neurological recovery beyond what would be expected from patient selection and spontaneous recovery, and adverse event rates (8.1% for SCS, 18.2% for DBS) indicate non-trivial surgical risk in this vulnerable population. Neurophysiological and neuroimaging biomarkers derived from EEG, functional near-infrared spectroscopy, functional MRI, and FDG-PET show promise for objective outcome prediction but await prospective validation. We conclude that DBS and SCS remain experimental, mechanistically motivated but not yet definitively proven therapies for chronic DOC, and that adequately powered randomized controlled trials, biomarker-guided patient selection, and dedicated ethical frameworks are needed before either modality can be recommended outside of research settings.

## 1 Introduction

Prolonged disorders of consciousness (DOC)—encompassing coma, the vegetative state/unresponsive wakefulness syndrome (VS/UWS), and the minimally conscious state (MCS)—represent one of the most challenging sequelae of severe acquired brain injury, imposing profound medical, social, and economic burdens on patients, families, and health systems (Othman et al., 2025). Although a minority of patients recover spontaneously within the first 6–12 months after injury, many remain in a chronic state of impaired arousal or awareness, for which no disease-modifying pharmacological therapy has been definitively established (Schnakers and Monti, 2017).

Pharmacological approaches such as amantadine have shown the ability to accelerate functional recovery, and zolpidem has produced transient paradoxical arousal in a subset of patients, but these effects are inconsistent and do not restore full consciousness in most cases (Schnakers and Monti, 2017). This therapeutic gap has motivated growing interest in neuromodulatory strategies capable of directly modulating the residual neural circuitry that supports arousal and awareness, including non-invasive techniques (transcranial direct current stimulation, transcranial magnetic stimulation, low-intensity focused ultrasound) and invasive approaches such as deep brain stimulation (DBS) and spinal cord stimulation (SCS) (Vitello et al., 2025).

Among invasive neuromodulation strategies, DBS of central thalamic and related structures, and cervical SCS, have accumulated the longest clinical track record, dating back more than four decades, and have recently been the subject of several systematic reviews and individual patient data (IPD) meta-analyses (Mattogno et al., 2017). Reflecting this uncertainty, contemporary clinical neurophysiology reviews note that the efficacy of both invasive and non-invasive brain stimulation trials for prolonged DOC remains a matter of active debate, with significant behavioral improvements still considered largely anecdotal (Ragazzoni et al., 2017). The evidence base remains fragmented across small, heterogeneous case series, and the field continues to debate patient selection criteria, optimal targets and stimulation parameters, and the extent to which observed improvements exceed the natural history of spontaneous recovery (Liao and Gao, 2026).

This narrative review synthesizes the past decade of literature (2016–2026) on DBS and SCS for the treatment of chronic DOC, with the following aims: (i) to summarize the neurobiological rationale underlying thalamic and spinal neuromodulation; (ii) to critically appraise the clinical evidence for DBS and SCS, including case series, cohort studies, and IPD meta-analyses; (iii) to synthesize neurophysiological and neuroimaging biomarkers that inform patient selection and mechanism; (iv) to compare the relative efficacy, safety, and practical considerations of the two modalities; and (v) to identify methodological gaps and propose directions for future research, in accordance with the need for both clinical optimism and scientific rigor voiced throughout this literature (Othman et al., 2025; Mattogno et al., 2017). As a narrative review, this work does not employ a formal systematic search strategy with pre-registered inclusion/exclusion criteria or quantitative meta-analytic pooling; rather, it integrates findings across the clinical, mechanistic, and biomarker literatures to provide a critical, conceptually organized synthesis, with particular attention to methodological limitations and causal inference.

## 2 Neurobiological rationale: the mesocircuit hypothesis and thalamocortical dysfunction

The rationale for thalamic neuromodulation in DOC rests principally on the ‘mesocircuit hypothesis’ articulated by Schiff, which proposes that multifocal brain injury produces widespread neuronal death and de-afferentation, leading to reduced excitatory drive onto the central thalamus, and downstream, hypoactivity of the anterior forebrain mesocircuit (the connections linking the central thalamus, striatum, globus pallidus, and frontal cortex) (Schiff, 2023). According to this model, a partially preserved but functionally silent frontoparietal network can, in principle, be reactivated by increasing thalamic output, providing a rationale for both central thalamic DBS and, indirectly, for ascending activation via cervical SCS (Yang et al., 2024).

Direct electrophysiological support for this framework has come from intraoperative microelectrode recordings. Single-unit recordings from the thalamus and overlying parietal cortex of patients with VS/UWS and MCS revealed markedly fewer active thalamic neurons in VS/UWS than in MCS, along with reduced thalamocortical cross-frequency coupling, findings that are broadly consistent with a graded loss of thalamocortical drive across the DOC spectrum, albeit based on only three patients (Magrassi et al., 2018). In a larger series of 23 patients undergoing central thalamic DBS, electrophysiological features of the centromedian-parafascicular (CM-pf) complex—particularly theta-band oscillatory dynamics, firing rates, and gamma-band synchronization—correlated with both baseline diagnosis and post-stimulation outcome, providing further support for the mesocircuit model and identifying candidate physiological biomarkers of recovery potential (He et al., 2023; Zhang et al., 2025).

Preclinical evidence provides more direct causal support. In a nonhuman primate model, central thalamic DBS applied during anesthesia-induced unconsciousness acutely restored arousal, increased functional MRI activity in prefrontal, parietal, and cingulate cortices, reinstated a broad dynamic repertoire of resting-state activity previously identified as a signature of consciousness, and restored a hierarchical cortical response to auditory novelty—effects that were absent when a control site in the ventrolateral thalamus was stimulated, arguing for anatomical specificity of the central thalamic target (Tasserie et al., 2022).

Collectively, these mechanistic studies provide converging, though largely correlational and small-sample, support for a causal role of central thalamic circuitry in the recovery of consciousness, and they justify the choice of central thalamic and CM-pf targets that dominate the clinical DBS literature reviewed below. A notable strength of this line of work is the combination of computational modelling, invasive human electrophysiology, and translational nonhuman primate causal manipulation, which is unusual in the DOC field and lends the mesocircuit hypothesis more mechanistic weight than most alternative accounts. Nonetheless, the model remains only partially testable in patients—most human electrophysiological data derive from patients already undergoing DBS surgery, introducing selection bias toward patients believed to have sufficient residual anatomy—and the relationship between mesocircuit re-activation and durable, rather than transient, recovery of consciousness remains to be established with longitudinal, multimodal recordings (He et al., 2023; Zhang et al., 2025).

The contrast between intact thalamocortical and basal-ganglia loops in physiological consciousness and the mesocircuit failure that characterizes pathological DOC is summarized in Figure 1.

**Figure 1:**
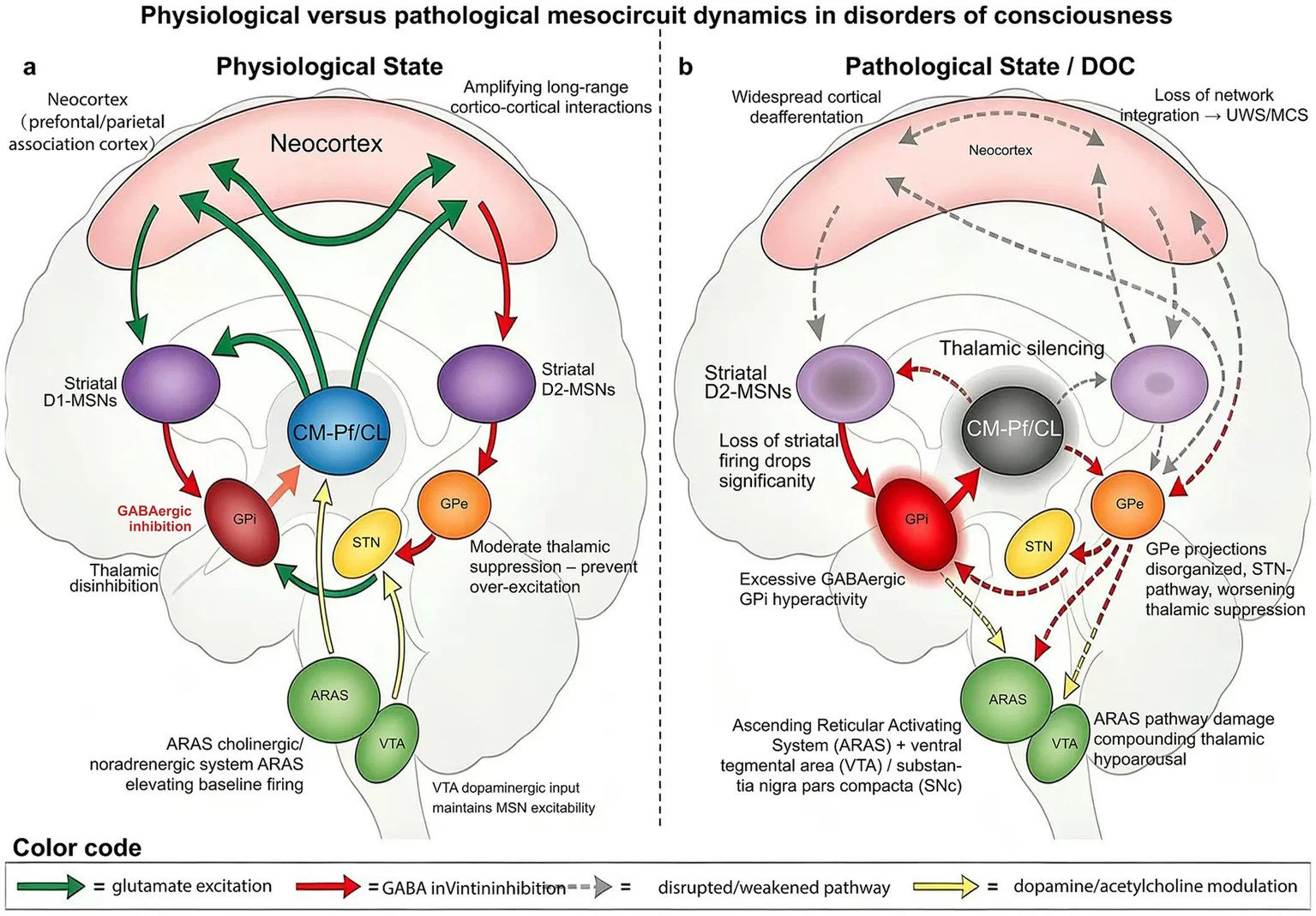
Schematic of circuit changes in physiological consciousness and pathological disorders of consciousness (DOC). **(a)** Physiological state: Intact cortico-cortical and basal-ganglia-thalamocortical loops support awareness. Glutamatergic neocortical output (green) drives striatal medium-spiny neurons (MSNs); balanced GABAergic inhibition (red) prevents thalamic over-excitation. Ascending reticular activating system (ARAS)/ventral tegmental area (VTA) neuromodulation (yellow) sustains neuronal activity. **(b)** Pathological DOC: Widespread cortical deafferentation and network disconnection underlie unresponsive wakefulness syndrome (UWS)/minimally conscious state (MCS). Striatal firing declines, globus pallidus internus (GPi) hyperactivity causes excessive GABAergic inhibition and thalamic silencing. Disorganized basal-ganglia circuits and ARAS/VTA damage aggravate thalamic hypoarousal. Grey dashed lines: disrupted/weakened pathways. Color code: green = glutamate excitation; red = GABA inhibition; yellow = dopamine/acetylcholine modulation; grey dashed = impaired pathways. This conceptual schematic was generated using Gemini 2.1-Pro (Google Gemini Advanced) and was edited and annotated by the authors.

## 3 Deep brain stimulation for disorders of consciousness

### 3.1 Targets, surgical technique, and patient selection

The centromedian-parafascicular complex (CM-pf) of the thalamic intralaminar nuclei and the broader central thalamus are the most frequently targeted structures for DBS in DOC, reflecting their central position within the mesocircuit and their established role in arousal regulation (Cao et al., 2024). A systematic review of 21 studies comprising 146 DOC patients treated with DBS between 1968 and 2023 found that the CM-pf and central thalamus were the most common targets, with stimulation frequencies predominantly set at 25 or 100 Hz, pulse widths of approximately 120 μs, and voltages of 0–4 V individually titrated to patient response; the pooled clinical efficacy rate across these heterogeneous studies was 39.7% (Cao et al., 2024).

Accurate targeting of the CM is technically demanding because the nucleus is poorly visualized on routine MRI. Two recent methodological studies addressed this limitation using different strategies. One study developed and validated machine-learning models (linear regression, k-nearest neighbor, support vector regression, and a deep neural network) trained on standard T1-weighted MRI to predict CM stereotactic coordinates, achieving a mean Euclidean error as low as 0.88–1.12 mm in healthy and epilepsy-patient datasets, respectively, with the best-performing deep neural network, thereby reducing dependence on advanced imaging and potentially improving access to CM-DBS in resource-limited settings (Singh et al., 2026). A complementary study refined indirect targeting coordinates by explicitly accounting for third-ventricular width, reducing the average targeting error from 4.40 ± 1.05 mm with conventional coordinates to 1.24 ± 0.5 mm with the proposed correction, with 84% of the estimated electrode contact volume falling within the CM versus 52% previously (Singh et al., 2025). Together, these two studies—one data-driven and one anatomically derived—converge on the same clinical problem (imprecise indirect CM targeting) and offer complementary, mutually reinforcing solutions, illustrating a maturing methodological subfield even though neither has yet been validated specifically in DOC cohorts (both were validated in epilepsy or healthy populations, a limitation that should be addressed before their routine adoption in DOC surgery).

For patients with the additional challenge of severe cerebral deformation—common after traumatic brain injury or hydrocephalus—conventional atlas-based planning is frequently inaccurate. A case series of four patients with chronic DOC and marked brain deformation (including two with significant hydrocephalus) demonstrated the feasibility of a robot-assisted, non-rigid thalamic registration technique for CM-pf targeting, achieving electrode placement within 0.4 mm of the planned target and producing a median 7-point increase in Coma Recovery Scale-Revised (CRS-R) scores postoperatively without surgical complications (Liu et al., 2026). While encouraging, the authors themselves caution that such improvements in a small, uncontrolled case series cannot be firmly distinguished from spontaneous recovery, underscoring a limitation that recurs throughout this literature.

Beyond targeting accuracy, structural neuroimaging offers insight into the downstream consequences of successful stimulation. Volumetric MRI analysis of five DOC patients who underwent unilateral CM-pf DBS, followed for up to 7 years, showed significant increases in limbic and paralimbic cortical volumes (parahippocampal and cingulate gyrus, insula) and subcortical structures (caudate, hippocampus, amygdala) coincident with functional recovery, raising the possibility that DBS-associated network reactivation may be accompanied by structural neuroplasticity, although the study could not determine whether these volumetric changes were a cause or a consequence of consciousness recovery (Raguž et al., 2021).

### 3.2 Clinical evidence: from early case series to individual patient data meta-analyses

Clinical experience with thalamic DBS for DOC dates back more than 40 years, but rigorous, prospective, multi-institutional data have only recently become available. The prospective multi-institutional CATS study registered three patients (two VS, one MCS) who underwent bilateral thalamic DBS and were followed for 18–48 months; all three showed increased EEG desynchronization/power spectrum changes and improved CRS-R scores, together with reduced spasticity, but none regained fully conscious behavior, illustrating the frequent dissociation between neurophysiological and clinically meaningful behavioral improvement that recurs across this literature (Magrassi et al., 2016). Editorial commentary accompanying this study emphasized the historical importance but modest clinical yield of thalamic DBS trials to date (Eskandar, 2018).

A larger single-center series from Zagreb selected 14 of 49 screened VS/MCS patients for CM-pf DBS using a strict multimodal battery (evoked potentials, PET, MRI); two of four MCS patients regained full consciousness with independent living, one further MCS patient regained consciousness while remaining wheelchair-bound, and one of ten VS patients improved to a conscious, command-following state, while three VS patients died of medical complications and seven remained without substantial improvement (Chudy et al., 2018). The same group’s subsequent ten-year, 83-patient experience (36 candidates, 32 implanted: 27 UWS, 5 MCS) reported improvement in seven patients, with three of five MCS patients emerging to full awareness and two becoming independent, versus only four of 27 UWS patients showing improvement (two reaching full awareness); the authors explicitly recommended DBS earlier in the clinical course, before irreversible musculoskeletal contractures develop, and called for multicenter collaboration to overcome persistent small sample sizes (Chudy et al., 2023).

This pattern—consistently better DBS responsiveness in MCS than in VS/UWS—recurs across the literature. A systematic review pooling 78 individual patients from 19 articles published since 1968 concluded that there is no unambiguous evidence that DBS can restore both consciousness and communication ability in DOC, and that in patients who did benefit, the contribution of spontaneous recovery could rarely be excluded given inconsistent time-from-injury reporting; the authors additionally raised substantive ethical concerns, including the risk of increasing patients’ self-awareness of their own limitations without net improvement in wellbeing, and the complexities of proxy consent in this incapacitated population (Vanhoecke and Hariz, 2017). A subsequent systematic review focused specifically on MCS following traumatic brain injury identified only eight studies (ten patients) with highly variable time-to-stimulation (3–252 months) and follow-up (10–120 months); seven of ten patients improved on validated outcome measures, but the review concluded that evidence remains insufficient given small, heterogeneous samples and the confound of spontaneous recovery within the first year (Rezaei Haddad et al., 2019). A broader narrative review of DBS for consciousness and cognitive impairment after traumatic brain injury reached similar conclusions while cataloguing an expanding set of candidate targets (ascending reticular activating system components, thalamus, fornix, nucleus accumbens, internal capsule, and brainstem nuclei) for both arousal and higher cognitive domains (Kundu et al., 2018).

The most methodologically rigorous evidence to date derives from two independent individual patient data (IPD) meta-analyses published in 2025–2026. The first pooled IPD from 49 patients (37 VS, 12 MCS) across seven centers who underwent central thalamic DBS; seven of 49 patients (14%) significantly recovered awareness, and in every case implantation occurred within 12 months of injury, with younger age and shorter injury-to-implantation interval predicting greater CRS-R improvement; the authors concluded that although cyclic central thalamic DBS acutely improved daytime awareness, there was insufficient evidence that it altered the natural history of neurological recovery in chronic DOC (Bergeron et al., 2025). A second, overlapping IPD meta-analysis pooling nine studies and 81 DBS-treated patients reported a pooled mean CRS-R improvement of 2.15 points (95% CI 1.06–3.23)—a modest effect—with only 39.5% of patients achieving the minimum clinically important difference of ≥three CRS-R points and 30.9% transitioning to a higher DOC category; heterogeneity across studies was substantial (I^2^ = 76.5%), etiology was largely non-predictive (although hypoxic–ischemic injury showed marginally better response), and adverse events including infections and seizures were reported (Lee et al., 2026). Read together, these two IPD meta-analyses—despite differing in inclusion criteria and precise pooled estimates—converge on a consistent, cautious message: DBS produces real but modest and highly heterogeneous improvements in a minority of carefully selected patients, predominantly those implanted early after injury, and neither study provides evidence that DBS reliably restores full consciousness or alters long-term recovery trajectories beyond what would be expected from patient selection and natural history.

In apparent contrast to the modest pooled effects above, a large single-center retrospective cohort of 365 consecutively admitted DoC patients (2011–2021) reported that 32.4% (12/37) of DBS-treated patients achieved improved consciousness at 1 year, versus only 4.3% (14/328) of conservatively managed patients, yielding an adjusted odds ratio of 11.90 (95% CI 3.65–38.46) after multivariate adjustment, with a significantly greater treatment effect in MCS than VS/UWS patients (Yang et al., 2023). This considerably larger effect size than that reported by the IPD meta-analyses likely reflects a combination of factors that merit critical scrutiny: retrospective, non-randomized treatment allocation (DBS candidates were presumably pre-selected for more favorable prognostic features), a single-center design without the between-center heterogeneity that inflated I^2^ in the pooled analyses, and possible residual confounding despite statistical adjustment. This discrepancy exemplifies a broader methodological tension in the DBS-DOC literature between retrospective cohort studies—prone to indication bias favoring surgically selected, better-prognosis patients—and IPD meta-analyses of largely uncontrolled case series, neither of which can substitute for a randomized, sham-controlled trial; the authors of the cohort study themselves called for randomized controlled trials (RCTs) to confirm their findings (Yang et al., 2023).

Advancing beyond aggregate outcome statistics, a multicenter analysis of 40 DBS-treated DOC patients targeting the CM-pf complex found that consciousness improvement was associated with better-preserved striatal gray matter and with electric field distributions extending below the centromedian nucleus into the inferior parafascicular nucleus and adjacent ventral tegmental tract—a brainstem-hypothalamus connecting pathway—and that the associated network overlapped with independently derived brain networks implicated in arousal-impairing stroke and awareness-impairing seizures, offering a principled, network-based framework for refining patient selection and electrode placement beyond anatomical targeting alone (Warren et al., 2025). At the single-patient mechanistic level, magnetoencephalography in one patient with prolonged MCS demonstrated that lower-frequency (50 Hz), larger-volume central thalamic DBS produced stronger increases in functional connectivity and neural variability than higher-frequency (130 Hz), smaller-volume stimulation, and was associated with clinically meaningful gains (improved visual pursuit, reduced spasticity, improved swallowing) 8 years after injury, even though all neurophysiological markers remained below healthy-control levels—a finding that nuances the simple assumption that higher stimulation frequency is uniformly superior and underscores the importance of individualized parameter optimization (Arnts et al., 2022).

Placing DBS in context with SCS, the largest systematic review and IPD analysis to date pooled 608 cases from 20 studies: five studies (92 patients) reported DBS outcomes, with an overall effectiveness rate of 40% (26% for VS, 74% for MCS), and adverse events in 18.2% of DBS-treated patients (comparable overall efficacy magnitude to SCS, discussed below, but with a notably higher complication rate) (Wu et al., 2023).

### 3.3 Neurophysiological and imaging biomarkers of response

Because behavioral assessment with the CRS-R is subjective, time-consuming, and can be confounded by motor impairment, several groups have sought objective neurophysiological or imaging correlates of DBS-induced consciousness change. Functional near-infrared spectroscopy (fNIRS) studies have been particularly productive, likely reflecting their bedside feasibility in this population. A brain hemodynamic transition-state analysis in 11 patients found that increases in a global ‘transition acceleration’ index of frontal and parietal information transmission correlated strongly with CRS-R improvement after DBS (R^2^ = 0.732), suggesting fNIRS-derived network dynamics as a candidate functional biomarker (Shu et al., 2023a). A complementary fNIRS functional-connectivity study of ten patients found that global communication efficiency tracked CRS-R change (R^2^ = 0.483) and that patients with traumatic etiology showed larger CRS-R gains than those with hemorrhagic or brainstem-infarction causes, again highlighting etiology as a potential effect modifier (Shu et al., 2023b). A more recent dynamic temporal-spectral fNIRS analysis of 13 patients similarly linked increased global and regional functional variability—particularly in the right hemisphere—to consciousness improvement after DBS (Lu et al., 2024).

An important caveat to interpreting these fNIRS findings as neural biomarkers is that fNIRS measures a hemodynamic (blood-oxygenation) signal, and its validity as a proxy for neuronal activity rests on the assumption of intact neurovascular coupling. In chronic DOC patients with widespread structural injury, deafferentation, and often significant cerebrovascular dysregulation, whether neurovascular coupling remains intact is generally neither tested nor reported in the cited studies. The observed hemodynamic changes could, in principle, reflect alterations in cerebral blood flow autoregulation or vascular reactivity rather than—or in addition to—underlying neuronal activity. This concern is reinforced by the autonomic findings reviewed below: heart rate variability (HRV) changes after DBS (Cao et al., 2025) indicate that stimulation can modulate autonomic and vascular tone, raising the possibility that at least a portion of the fNIRS signal reflects vascular or autonomic reactivity rather than purely neural activation. Future fNIRS studies in DOC should ideally incorporate concurrent measures of cerebral blood flow or arterial spin labeling to disentangle neural from vascular contributions, and should explicitly assess neurovascular coupling integrity in their patient cohorts.

EEG-based studies provide independently correlated evidence at a different spatial–temporal scale, though it is important to qualify that no study to date has performed simultaneous fNIRS-EEG recording in the same DOC patients to directly test whether the two modalities track a common underlying process. In nine MCS patients, 100 Hz DBS increased whole-brain and local EEG functional connectivity and improved network small-world properties compared with sham stimulation, with sustained CRS-R gains observed in three patients maintained on chronic 100 Hz stimulation for 6 months (Dang et al., 2023a). The closest approximation to cross-modal convergence is the 70 Hz frequency finding, where an fNIRS study (Si et al., 2018) and an EEG study (Zhuang et al., 2024) reached convergent conclusions in independent patient cohorts, but not via concurrent recording. Thus, fNIRS and EEG should be regarded as two independently correlated modalities rather than a validated joint biomarker; simultaneous fNIRS-EEG recording represents an important and currently unaddressed future direction.

Sleep architecture represents an additional, underexplored physiological domain: longitudinal polysomnographic follow-up of a patient with chronic MCS receiving central thalamic DBS (CT-DBS) demonstrated late and progressive alterations in sleep dynamics emerging only after prolonged stimulation, suggesting that some DBS-induced neurophysiological reorganization may unfold over a substantially longer timescale than the days-to-months windows captured by most fNIRS/EEG studies reviewed above, and that sleep-stage architecture could serve as a complementary, slow-timescale biomarker of network-level change (Adams et al., 2016). Beyond central measures, autonomic nervous system monitoring via heart rate variability (HRV) in eight patients found that 100 Hz DBS produced the most pronounced HRV changes and that high-frequency, low-frequency, and total HRV power showed prognostic value, suggesting that autonomic biomarkers may provide an additional, easily-acquired readout of central neuromodulatory effect (Cao et al., 2025).

Cerebral metabolic imaging with ^18^F-fluorodeoxyglucose positron emission tomography (FDG-PET) constitutes a well-established modality in the broader DOC literature—particularly for differentiating VS/UWS from MCS and for identifying preserved metabolic networks that may predict recovery potential—but has been notably underutilized as a biomarker of DBS/SCS treatment response. In the DBS literature reviewed here, FDG-PET appears only as a component of the multimodal patient-selection battery in the Zagreb series (Chudy et al., 2018), where it was used alongside evoked potentials and MRI to screen candidates, but was not employed as a pre- or post-stimulation outcome measure. More broadly in DOC research, FDG-PET has demonstrated that preserved metabolic activity in the frontoparietal network and thalamus correlates with the level of consciousness and may predict late recovery, and that metabolic connectivity patterns can distinguish MCS from VS/UWS with high accuracy. The absence of FDG-PET from the DBS/SCS biomarker literature likely reflects the practical challenges of serial PET imaging in medically fragile, behaviorally impaired patients, as well as the relative recency of systematic biomarker development in this field. Incorporating pre- and post-stimulation FDG-PET—or hybrid PET-MRI—into future trials would provide a direct measure of cerebral glucose metabolism that complements the hemodynamic (fNIRS/fMRI) and electrophysiological (EEG) readouts currently dominant, and would help determine whether stimulation-induced behavioral gains are accompanied by sustained metabolic reactivation of thalamocortical networks.

Collectively, these biomarker studies share methodological strengths—objective, quantitative, repeatable measures that circumvent the ceiling and floor effects of behavioral scales—but also common limitations: sample sizes of 8–13 patients, single-center designs, heterogeneous stimulation protocols (frequencies ranging from 25 to 130 Hz across studies), an absence of independent validation cohorts, and, for fNIRS in particular, untested assumptions regarding neurovascular coupling integrity in chronically injured brains. A promising future direction is illustrated by a neural-field-theory-based computational framework that fits individualized EEG power-spectrum models to derive personalized stimulation waveforms designed to induce ‘healthy-like’ brain activity; while so far validated only in silico, this approach exemplifies a shift from empirical, one-size-fits-all parameter selection toward mechanistically grounded, patient-specific stimulation design, and merits prospective clinical validation (Polyakov et al., 2025).

## 4 Spinal cord stimulation for disorders of consciousness

### 4.1 Rationale, targets, and surgical technique

Cervical SCS was originally developed to increase cerebral blood flow and cortical arousal via ascending activation of the reticular formation-thalamus-cortex pathway, and has been applied to DOC for over three decades using epidural electrodes typically placed at the C2–C4 level (Yamamoto et al., 2017). It is important to emphasize that activation of the ascending reticular activating system, restoration of thalamocortical signaling, and mesocircuit reactivation by SCS remain plausible mechanistic hypotheses rather than established causal mechanisms in humans. The available human evidence consists primarily of associations between SCS-induced EEG, fNIRS, or fMRI changes and CRS-R improvement; these correlational findings do not establish causal mediation, and the precise pathways by which epidural cervical stimulation influences forebrain arousal circuits remain incompletely characterized. A review of the clinical SCS-DOC literature identified 19 eligible studies (532 cases), summarized further in the comparative section below (Piedade et al., 2023).

A key technical determinant of outcome is precise midline placement of the stimulating electrode array, since lateral deviation reduces bilateral activation. A retrospective single-center study of 69 DOC patients undergoing C2–4 SCS found that a novel C2–3 dural exposure procedure, performed with the patient in the lateral decubitus position, achieved complete midline electrode coverage in 93.8% of cases versus 54.7% with conventional technique (*p* = 0.004), without differences in operative time, bleeding, or infection, indicating that this refinement improves technical accuracy at no additional safety cost, although the study’s retrospective, non-randomized design and single-center setting limit generalizability (Dang et al., 2023b).

Beyond behavioral endpoints, resting-state and stimulation-triggered functional MRI have been used to characterize network-level effects of epidural high-cervical SCS. In 21 patients with chronic DOC and severe spasticity treated with tonic or ‘Burst’ stimulation modes, CRS-R scores improved significantly (*p* = 0.028) in 38.1% of patients, spasticity improved in 52.4%, and fMRI performed in a subset of nine patients showed increased functional connectivity of the right anterior insula with the dorsal attention, visual, and default mode networks after stimulation—providing early network-level correlates of clinical improvement, though the correlational, non-blinded design and small imaging subsample warrant caution (Vorobyev et al., 2023).

### 4.2 Clinical evidence and predictors of outcome

The largest dedicated review of cervical SCS for DOC pooled 19 peer-reviewed clinical studies comprising 532 cases; overall, 255 patients (47.9%) were classified as responsive to stimulation. Restricting to studies published after MCS was formally defined as a diagnostic entity, 402 patients were in UWS and 113 in MCS; responsiveness was reported in 170 of 402 UWS patients (42.3%) and 78 of 113 MCS patients (69.0%), again demonstrating the now-familiar pattern of greater efficacy in MCS than UWS/VS, although the authors caution that criteria for ‘responsiveness’ varied substantially across the pooled studies, limiting direct comparability (Piedade et al., 2023).

Prognostic factor analysis has been a recurring focus of the Chinese SCS-DOC literature. In an early series of 12 VS patients treated with cervical SCS, mean CRS-R scores improved from 6.25 at baseline to 10.8 at last follow-up (11.1 months); five patients achieved a responsive outcome (three fully recovered, two evolved to MCS). Presence of the N20 somatosensory evoked potential component and multifocal CT/MRI/PET abnormalities predicted better outcome, whereas an ischemic-anoxic injury etiology predicted poorer outcome (Xu et al., 2019). A much larger subsequent cohort of 110 patients found an overall positive-outcome rate of 31.8% (35/110), with MCS patients showing a 45.53-percentage-point higher improvement rate than VS/UWS patients (statistically significant), younger age (≤19 years) and MCS diagnosis independently associated with positive outcome in multivariate analysis, and a nomogram incorporating age, consciousness state, and etiology achieving good discrimination (c-index 0.794) (Yang et al., 2022). These prognostic factors—consciousness level at baseline, age, injury-to-treatment interval, and evoked-potential/imaging integrity—recur across both the SCS and DBS literatures, suggesting a shared, etiology-independent substrate of ‘recoverability’ that could inform unified selection algorithms across both neuromodulation modalities.

More recent series report additional evidence of clinical benefit on functionally meaningful, non-CRS-R endpoints. A retrospective cohort of 81 tracheotomized brain-injury patients with DOC found that short-term SCS significantly increased successful tracheal decannulation compared with standard care (50.0% vs. 25.7%, χ^2^ = 5.24, *p* = 0.022), extending the therapeutic rationale for SCS beyond consciousness level per se to include swallowing and airway protection, functions of substantial practical importance for nursing burden and quality of life, although the retrospective design and lack of blinding again limit causal inference (Huang et al., 2025). In parallel, EEG- and ERP-based studies have reinforced behavioral findings: in 27 patients undergoing SCS, CRS-R scores improved significantly at 3 and 6 months post-surgery (*p* < 0.001), accompanied by improvements in resting-state EEG power in the alpha and beta bands and in P300 event-related potential amplitude, peak, and latency, all surviving correction for multiple comparisons in the higher-frequency bands (Sun et al., 2024). Complementary evidence from a study using EEG source-space network analysis in 31 chronic DoC patients found that sustained increases in alpha/beta-band network energy across five canonical resting-state networks, peaking at 1 month post-implantation, and consolidation of beta-band cross-network communication, were the neurophysiological features most strongly associated with favorable 6-month CRS-R outcomes, providing an early (1-week to 1-month) electrophysiological readout that could in principle guide continuation or cessation of a therapeutic SCS trial before committing to prolonged implantation (Zhang et al., 2026). A separate fMRI biomarker study found that thalamic amplitude of low-frequency fluctuations (ALFF) correlated with 6-month post-SCS CRS-R improvement in both traumatic (r = 0.64) and cerebrovascular (r = 0.59) DoC etiologies, and identified anterior cingulate-thalamic ALFF as a cross-etiology candidate biomarker for guiding SCS target selection (Qin et al., 2025).

Mechanistically, short-term SCS (20 min) has been shown to acutely increase cortical connectivity as measured by a genuine permutation cross mutual information metric, with connectivity changes in frontal, parietal, and fronto-parietal/fronto-occipital regions correlating with CRS-R change (r = 0.66–0.80); notably, short-term (20-min) stimulation alone did not significantly change CRS-R scores, whereas 6 months of continued SCS did, indicating that acute electrophysiological engagement is a necessary but not sufficient marker of eventual clinical benefit and that a minimum treatment duration is likely required before behavioral gains emerge (Aihemaitiniyazi et al., 2026). This latter point is important for clinical trial design, since studies assessing behavioral outcomes only immediately after brief test stimulation risk underestimating the therapeutic potential of SCS, while studies using only long-term outcomes cannot dissociate treatment effect from spontaneous recovery over the same interval.

In the previously introduced 608-case systematic review and IPD analysis, ten studies (508 patients) reported SCS outcomes for DoC overall, with an estimated effectiveness rate of 37%; disaggregated by consciousness level, the effectiveness rate was 30% for VS (five studies, 343 patients) and 63% for MCS (three studies, 53 patients)—again confirming superior SCS responsiveness in MCS relative to VS/UWS, mirroring the DBS pattern. The overall adverse-event rate for SCS was 8.1%, markedly lower than the 18.2% adverse-event rate reported for DBS in the same analysis, suggesting a more favorable risk profile for the less invasive cervical epidural approach, though this comparison is based on indirect, non-randomized pooling across different study populations and should not be over-interpreted as a head-to-head safety comparison (Wu et al., 2023).

### 4.3 Stimulation parameters and mechanistic insights

Because the SCS parameter space (frequency, inter-stimulus interval, pulse width, electrode configuration, and contact-level programming) is large and empirically under-explored, several groups have systematically compared stimulation frequencies using EEG and fNIRS readouts. In 11 MCS patients, resting-state EEG recorded before and after single-session SCS at five frequencies (5, 20, 50, 70, and 100 Hz) showed significantly altered relative power and synchronization in the delta and gamma bands specifically at 5, 70, and 100 Hz, with no significant EEG changes at 20 Hz, 50 Hz, or sham stimulation—demonstrating that SCS effects on cortical activity are frequency-specific rather than generically present at any stimulation setting (Bai et al., 2017). Consistent with this, an fNIRS study of ten patients found significantly greater prefrontal hemodynamic responses at 70 Hz and 100 Hz than at 50 Hz, and significantly improved prefrontal-occipital functional connectivity specifically at 70 Hz, which the authors interpreted as evidence of frequency-dependent engagement of the reticular formation-thalamus-cortex pathway and proposed 70 Hz as a candidate optimal frequency (Si et al., 2018). This proposal received independent behavioral-EEG corroboration in a study of 23 patients undergoing short-term SCS implantation, in which parietal-occipital beta-band connectivity changes 1 week after 70 Hz stimulation correlated with 3-month CRS-R change, again converging on 70 Hz and posterior beta-band connectivity as a mechanistically coherent and reproducible signature across at least three independent patient cohorts (Zhuang et al., 2024).

Notably, pulse width is conspicuously under-reported in the SCS-DOC literature. In contrast to the DBS section, where pulse widths of approximately 120 μs are consistently reported (Cao et al., 2024), none of the SCS studies reviewed here explicitly state the pulse width used, and the parameter is not listed in the comparative Table 1 for any SCS study. This represents a significant reporting gap in the source studies: pulse width is a critical determinant of the volume of tissue activated and of neural recruitment patterns in SCS, and its omission precludes systematic comparison across studies and meta-analytic pooling of parameter–outcome relationships. We explicitly flag this as an under-explored dimension of the SCS parameter space that should be standardized in future trial reporting.

**Table 1:** Key clinical studies of DBS and SCS for chronic disorders of consciousness (2016–2026).

Study	Modality	N (diagnosis)	Target/parameters	Follow-up	Outcome	Key findings
Magrassi et al. (2016)	DBS	3 (2 VS, 1 MCS)	Ant. intralaminar, bilat.; Hz NR	18–48 mo	CRS-R; EEG	CRS-R↑; none regained full consciousness
Chudy et al. (2018)	DBS	14 (10 VS, 4 MCS)	CM-pf; params NR	38–60 mo	Consciousness recovery	2/4 MCS independent; 1/10 VS command-responsive; 7 no gain
Chudy et al. (2023)	DBS	32 (27 UWS, 5 MCS)	CM-pf (L or better-preserved side)	10-y series	Consciousness level	Improved 7/32; MCS 3/5 emerged; UWS 4/27 improved
Yang et al. (2023)	DBS	37 DBS vs. 328 cons.	Target/params NR	1 y	Consciousness improvement	32.4% vs. 4.3%; MCS ≫ VS/UWS (interaction *p* < 0.001)
Arnts et al. (2022)	DBS	1 MCS	Central thalamus; 50 vs. 130 Hz	Chronic MCS (8 y post-injury)	MEG; clinical signs	50 Hz + larger VTA → ↑connectivity; objective awareness gain limited
Warren et al. (2025)	DBS	40 DoC	CM-pf (± inferior Pf / VTT)	NR	Consciousness recovery	Recovery linked to striatal GM + stimulation below CM
Bergeron et al. (2025)	DBS (IPD)	49 (37 VS, 12 MCS)	Central thalamus; multi-center	Variable	CRS-R	7/49 recovered awareness; all implanted ≤12 mo post-injury
Lee et al. (2026)	DBS (IPD)	81 DoC	Mixed thalamic targets	Variable	CRS-R	ΔCRS-R + 2.15; MCID ≥3 in 39.5%; I^2^ = 76.5%
Yamamoto et al. (2017)	SCS	31 (21 VS, 10 MCS)	C2–C4 epi.; 5 Hz, 5 min/30 min daytime	≤12 mo	Consciousness; motor	Recovered 3 VS + 7 MCS; all ABR-V + N20 and SCS ≤ 9 mo post-injury
Xu et al. (2019)	SCS	12 VS	C2–4 epi.; params NR	Mean 11.1 mo	CRS-R	6.25 → 10.8; 5/12 responsive (3 recovered, 2 MCS)
Yang et al. (2022)	SCS	110 DoC	Target/params NR	Long-term	Positive outcome	Overall 31.8%; MCS + 45.5% vs. VS/UWS; age ≤19 predictive
Huang et al. (2025)	SCS	46 stSCS vs. 35 ctrl	Short-term SCS; params NR	In-hospital	Tracheal decannulation	50.0% vs. 25.7% (*p* = 0.022)
Sun et al. (2024)	SCS	27 DoC	Cervical SCS; params NR	3–6 mo	CRS-R; EEG/P300	CRS-R↑; EEG α/β and P300 improved post-SCS
Zhang et al. (2026)	SCS	31 chronic DoC	Cervical SCS; params NR	EEG 1 wk./1 mo → CRS-R 6 mo	Source EEG; CRS-R	α/β NetPower↑ at 1 mo linked to 6-mo recovery
Aihemaitiniyazi et al. (2026)	SCS	DoC (SCS vs. sham)	Cervical; 20-min acute + chronic	6 mo	CRS-R; G_PCMI	20 min: no CRS-RΔ; 6 mo: CRS-R↑; connectivity–CRS-R r = 0.66–0.80
Wu et al. (2023)	Both (SR)	608 (SCS 508; DBS 92)	Mixed	Mixed	Effectiveness; AEs	SCS 37% (VS 30%/MCS 63%); DBS 40% (VS 26%/MCS 74%); AE 8.1%/18.2%

Heterogeneity in targets, parameters, follow-up, and endpoints limits cross-study pooling. AE, adverse event; ABR, auditory brainstem response; CM-pf, centromedian–parafascicular; cons., conservative care; CRS-R, Coma Recovery Scale–Revised; DoC, disorders of consciousness; epi., epidural; GM, gray matter; IPD, individual participant data; MCS, minimally conscious state; NR, not reported in source; SEP, somatosensory evoked potential; SR, systematic review; stSCS, short-term SCS; UWS, unresponsive wakefulness syndrome; VS, vegetative state; VTA, volume of tissue activated; VTT, ventral tegmental tract. Rows restricted to studies with extractable clinical endpoints; blank/NR cells illustrate reporting gaps that preclude quantitative synthesis.

Beyond frequency, the inter-stimulus interval (ISI) has also been shown to matter: in nine patients with implanted SCS devices, a shorter ISI (30 s bursts) produced greater prefrontal blood-volume increases than longer ISIs, an effect that was more pronounced in patients with a subsequently favorable prognosis, suggesting that acute fNIRS responsiveness to ISI manipulation could itself serve as an early prognostic test, although this requires validation in larger, prospectively defined cohorts (Zhang et al., 2018). Complementing frequency-domain findings, entropy-based analyses in 14 patients (7 MCS, 7 VS/UWS) found that permutation entropy of resting EEG increased during a 20-min SCS session and decreased when stimulation was switched off, with larger increases in MCS than VS/UWS patients, particularly over frontal regions—providing a complexity-based, information-theoretic complement to the power/connectivity metrics used elsewhere in this literature and again showing MCS patients as more electrophysiologically responsive than VS/UWS patients (Wang et al., 2020).

Electrode contact configuration and contact-level programming represent another under-investigated dimension of SCS parameter optimization. Most SCS-DOC studies report only the vertebral level of electrode placement (typically C2–C4) and whether midline coverage was achieved, without specifying which individual contacts were active, their polarity, or whether differential programming across contacts was employed. A recent report introduced a ‘differential target multiplexed SCS’ paradigm that interleaves distinct stimulation patterns across electrode contacts, borrowing programming strategies from chronic pain SCS, but provided no quantitative comparison of outcomes by contact level or laterality (Li et al., 2024). Given that lateral deviation from midline is known to reduce bilateral activation and that dorsal column somatotopy means that different contacts engage different ascending fiber populations, systematic investigation of contact-level and laterality effects on outcome is an important gap. Future studies should report active contact configurations, polarity, and pulse width as standard parameters, and should examine whether contact-level programming can be optimized on an individual patient basis.

Using transcranial magnetic stimulation combined with EEG (TMS-EEG) to directly probe cortical excitability and complexity, a study of 20 DOC patients (10 UWS, 10 MCS) found that, despite negligible change in CRS-R scores immediately after a single SCS session, the MCS subgroup showed significant increases in global mean field potential amplitude, the perturbational complexity index (PCIst), and TMS-evoked natural frequency, whereas the UWS subgroup showed no significant TMS-EEG changes; baseline CRS-R correlated with the magnitude of ΔPCIst (r = 0.67) and ΔNF (r = 0.72), reinforcing both the dissociation between acute physiological and behavioral change and the principle that baseline consciousness level predicts physiological responsiveness to a single stimulation session (Wang et al., 2023).

Because SCS implantation requires general anesthesia in patients who are already critically vulnerable, two studies examined the perioperative anesthetic period itself as both a safety and a mechanistic window. Intraoperative EEG recorded from ten MCS patients under general anesthesia during SCS implantation showed that patients with good one-year prognosis exhibited increased slow-oscillation and alpha-band power in frontal regions during the maintenance phase, whereas those with poor prognosis showed increased phase-amplitude modulation index alongside reduced connectivity diversity and reduced theta/delta network efficiency—suggesting that intraoperative EEG signatures of thalamocortical and cortico-cortical connectivity may have prognostic value obtainable even before the therapeutic effect of SCS itself can be assessed (Jin et al., 2023). Extending this line of work, a prospective non-randomized comparative-effectiveness trial in 34 DoC patients undergoing SCS implantation found that esketamine, compared with propofol, was associated with faster recovery of spontaneous respiration (12.0 vs. 17.4 min, *p* = 0.005), less need for intraoperative vasopressors (17.7% vs. 52.9%, *p* = 0.034), better-preserved EEG permutation entropy, and higher gamma-band functional connectivity during anesthesia maintenance, together with improved 3-month consciousness outcomes—raising the intriguing possibility that anesthetic choice during SCS implantation is not merely a safety consideration but may itself influence neurological outcome, a hypothesis that, given the non-randomized allocation based on clinical practice in this study, requires confirmation in a randomized trial before influencing standard practice (Qin et al., 2026).

## 5 Comparative evidence, combined approaches, and multimodal neuromodulation

Direct, within-study comparisons of DBS and SCS remain scarce, and most comparative inference is necessarily indirect, drawn from separate literatures pooled within broader systematic reviews. The 608-case systematic review and IPD analysis discussed above found broadly similar overall effectiveness for SCS (37%) and DBS (40%), with both modalities showing markedly better response in MCS (SCS 63%, DBS 74%) than in VS/UWS (SCS 30%, DBS 26%), but a more than twofold higher reported adverse-event rate for DBS (18.2%) than SCS (8.1%) (Wu et al., 2023).

Synthesizing the prognostic evidence across both literatures, the patient profile most consistently associated with favorable response comprises: (i) a diagnosis of MCS rather than VS/UWS; (ii) younger age; (iii) shorter injury-to-treatment interval (ideally within 12 months); and (iv) preserved neurophysiological and structural integrity (evoked potentials, striatal gray matter, thalamic metabolic activity). For DBS specifically, the IPD meta-analyses indicate that benefit is concentrated in patients implanted within 12 months of injury, and that hypoxic–ischemic etiology showed marginally better response in one analysis (Lee et al., 2026), though etiology was largely non-predictive overall. For SCS, the fNIRS study found that traumatic etiology was associated with larger CRS-R gains than hemorrhagic or brainstem-infarction causes (Shu et al., 2023b), while an earlier SCS series found that ischemic-anoxic etiology predicted poorer outcome (Xu et al., 2019). This apparent divergence in etiology effects between the DBS and SCS literatures should be interpreted with considerable caution: the DBS finding derives from a pooled IPD meta-analysis with heterogeneous etiologic classification, while the SCS findings come from individual cohort studies with small etiologic subgroups, and neither comparison is sufficiently powered to establish a genuine modality-by-etiology interaction. It is possible that the divergent findings reflect differences in patient populations, injury severity, or timing rather than a true difference in how the two modalities interact with etiology. Nonetheless, this discrepancy underscores that etiology should not be treated as a uniformly predictive or non-predictive factor across both modalities, and future trials should stratify enrollment by etiology to enable direct comparison.

Taken together with the differing invasiveness, cost, and reversibility of cranial versus spinal implantation, these figures suggest that, all else being equal, cervical SCS may present a more favorable risk–benefit ratio as an initial invasive neuromodulation strategy in appropriately selected DOC patients—particularly VS/UWS patients whose lower expected efficacy makes the additional risk of intracranial surgery harder to justify—whereas DBS may be considered in MCS patients with preserved thalamic and striatal anatomy who can access specialized centers with stereotactic expertise. However, this recommendation is necessarily tentative, being based on an indirect comparison across non-randomized, heterogeneous study populations rather than a head-to-head trial, and should not substitute for individualized, multidisciplinary case-by-case decision-making (Wu et al., 2023).

Key clinical studies of DBS and SCS for chronic disorders of consciousness published between 2016 and 2026 are summarized in Table 1. Figure 2 outlines how DBS of the CM-Pf/CL complex and cervical SCS at C2–C4 act on this mesocircuit to restore thalamocortical drive.

**Figure 2:**
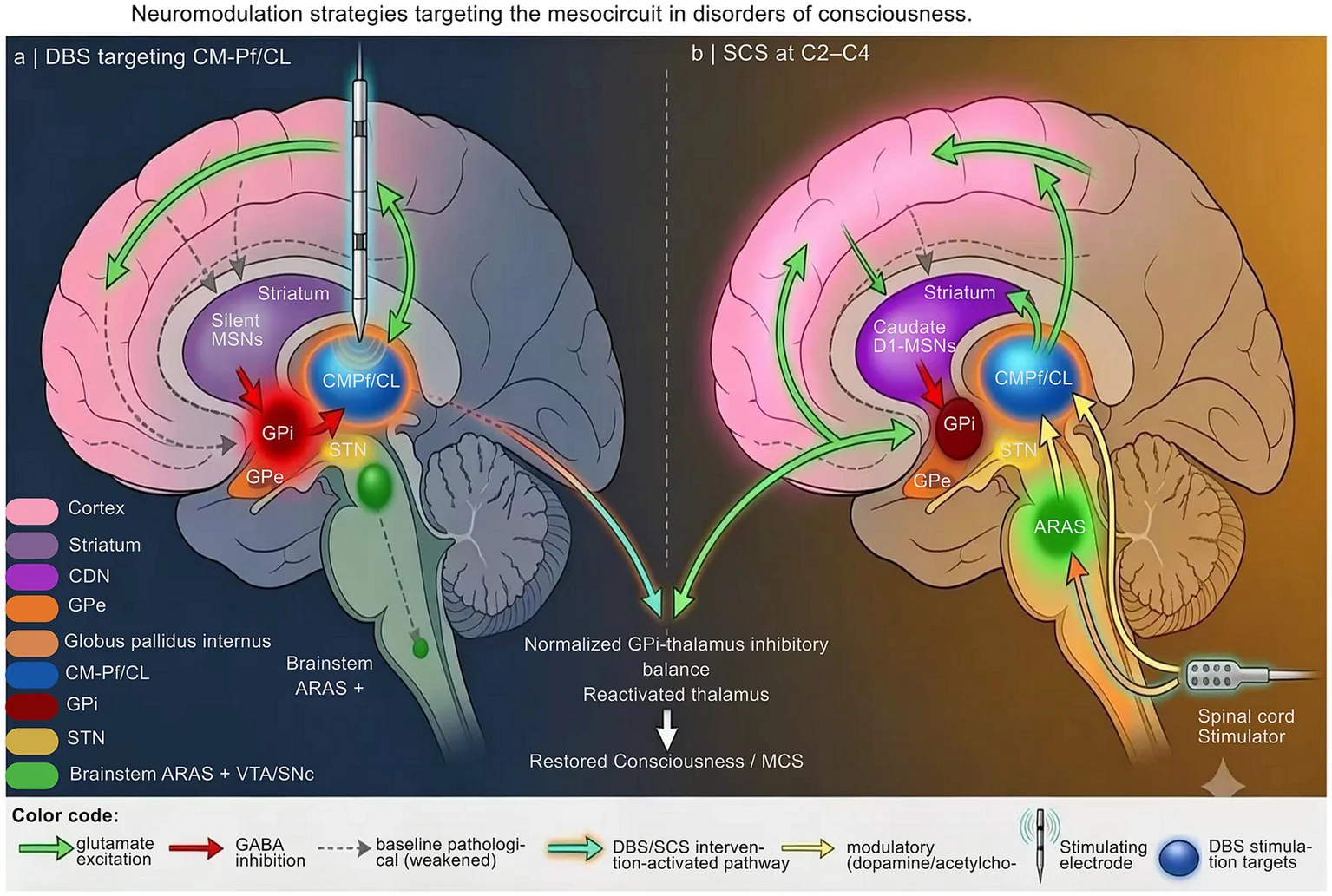
Neuromodulation interventions targeting the mesocircuit in disorders of consciousness. **(a)** Deep-brain stimulation (DBS) targeting CM-Pf/CL. DBS activates the thalamic CM-Pf/CL complex, counteracts pathological GPi-mediated inhibition, reactivates thalamic function, and restores GPi-thalamus inhibitory balance to promote consciousness recovery/minimally conscious state (MCS). **(b)** Spinal cord stimulation (SCS) at C2-C4. Cervical SCS boosts ascending reticular activating system (ARAS) output, enhances thalamocortical and striatal circuit activity, and re-establishes functional mesocircuit signaling. Color code: green, glutamate excitation; red, GABA inhibition; grey dashed, baseline pathological/weakened pathway; teal, DBS/SCS-activated pathway; yellow, dopamine/acetylcholine modulation; icon, stimulating electrode. This conceptual schematic was generated using Gemini 2.1-Pro (Google Gemini Advanced) and was edited and annotated by the authors.

A broader systematic review of 69 studies (58 human, 11 animal) spanning all neuromodulation modalities for DoC—including DBS (*n* = 15 human studies), transcranial magnetic stimulation (*n* = 18), transcranial direct-current stimulation (*n* = 15), spinal cord stimulation (*n* = 6), low-intensity focused ultrasound (*n* = 2), and median nerve stimulation (*n* = 2)—concluded that DBS appeared to produce greater improvements than other modalities, but that repetitive TMS also showed clinical potential with substantially lower invasiveness, and cautioned that clinical outcomes across all modalities were mixed and potentially confounded by concurrent pharmacological treatment and natural recovery (Dutta et al., 2025). This broader context is important: DBS and SCS should not be considered in isolation from the wider non-invasive neuromodulation armamentarium, and future trial design should ideally compare invasive approaches not only against no treatment/best supportive care but also against non-invasive alternatives with more favorable safety profiles, before recommending invasive surgery as a first-line neuromodulatory intervention (Dutta et al., 2025; Xia et al., 2018).

An emerging trend, illustrated by a novel differential target multiplexed SCS paradigm designed to interleave distinct stimulation patterns across electrode contacts, reflects ongoing efforts within the SCS literature to borrow programming strategies from other established SCS indications (e.g., chronic pain) and adapt them to the DOC population, a strategy of cross-pollination that may accelerate parameter optimization relative to the largely empirical frequency-sweep approach that has dominated the field to date (Li et al., 2024).

## 6 Critical appraisal: methodological quality, safety, and ethical considerations

Across both the DBS and SCS literatures reviewed above, several recurring methodological limitations constrain the strength of causal inference that can be drawn. First, nearly all studies are small (typically fewer than 30 patients per center) and uncontrolled or, at best, retrospectively controlled, with only two IPD meta-analyses approaching an adequately powered synthesis for DBS (Bergeron et al., 2025; Lee et al., 2026) and none identified for SCS; substantial between-study heterogeneity (I^2^ = 76.5% in one DBS meta-analysis) further limits the precision of pooled effect estimates (Lee et al., 2026).

Second, spontaneous recovery—which can occur up to and even beyond 12 months after traumatic injury—represents a pervasive and rarely fully excluded confound, and this limitation should be applied more consistently across the evidence base. Improvements observed in uncontrolled cohorts, particularly following relatively early implantation, cannot be causally attributed to stimulation alone. Several additional factors may contribute to observed gains: regression to the mean in patients selected because of low baseline scores; natural fluctuations in arousal and awareness that characterize the DOC trajectory; concurrent rehabilitation interventions that are often intensified around the time of implantation; medication changes (e.g., reduction of sedating agents, initiation of amantadine or other wakefulness-promoting drugs); and the selection of patients with favorable prognostic features for surgical candidacy, which creates indication bias that cannot be fully adjusted for statistically. The IPD meta-analyses have been commendably cautious on this point (Bergeron et al., 2025; Lee et al., 2026), but individual case series and retrospective cohorts frequently describe improvements as treatment effects without adequately addressing these alternative explanations. Only randomized, sham-controlled or adequately matched controlled designs can definitively disentangle stimulation-specific effects from the natural history and concurrent care.

Third, outcome measurement is dominated almost exclusively by the CRS-R, a validated but examiner-dependent behavioral scale that may under- or over-estimate covert awareness and cannot easily be blinded in unmasked case series, motivating the parallel development of quantitative EEG/fNIRS/fMRI biomarkers discussed in this review, though these too await prospective validation as primary trial endpoints (Cao et al., 2024). Fourth, stimulation parameters (frequency, target, duty cycle, pulse width, contact configuration) are highly heterogeneous across studies and are rarely justified by a pre-specified rationale, complicating comparison and meta-analytic pooling (Cao et al., 2024).

Regarding safety, pooled adverse-event rates of 8.1% for SCS and 18.2% for DBS in the largest available comparative analysis indicate that both procedures carry non-trivial surgical risk in an already medically fragile population, including reported infections and seizures with DBS (Lee et al., 2026; Wu et al., 2023). Because DOC patients cannot meaningfully participate in informed consent, all surgical and stimulation-related risk must be weighed by surrogate decision-makers under considerable uncertainty regarding the probability and nature of benefit, a burden explicitly discussed in ethical analyses of this literature (Vanhoecke and Hariz, 2017).

The ethical dimension of DBS and SCS trials in DOC extends well beyond standard surgical risk–benefit calculus and warrants more detailed consideration. As highlighted in a dedicated systematic review of cases and ethics, a particularly difficult scenario is the possibility that neuromodulation partially restores awareness without restoring functional independence or the capacity for meaningful communication, potentially increasing patients’ own awareness of pain, dependency, or diminished quality of life without a compensating gain in agency or wellbeing—an outcome that some ethicists argue could constitute harm rather than benefit, despite improved CRS-R scores (Vanhoecke and Hariz, 2017). Several additional ethical dimensions deserve explicit attention. First, surrogate authorization: because patients lack decision-making capacity, consent must be obtained from legally authorized representatives, who must weigh uncertain benefit against surgical risk without direct knowledge of the patient’s current subjective experience; ideally, decisions should incorporate any previously expressed patient preferences (e.g., advance directives, prior values discussions) to the extent these are available and applicable. Second, proportionality of surgical risk: the 18.2% adverse-event rate for DBS and 8.1% for SCS must be weighed not only against the probability of consciousness gain but also against the likelihood of partial recovery that may increase suffering without restoring agency. Third, criteria for stopping stimulation: there is currently no consensus on when a therapeutic trial should be deemed unsuccessful and stimulation discontinued, particularly given evidence that some neurophysiological and behavioral changes emerge only after months of stimulation (Adams et al., 2016); clear, pre-specified stopping rules should be incorporated into trial protocols. Fifth, device maintenance and explanation: implanted pulse generators require battery replacement and may malfunction, and the question of whether and when to explant a device in a patient who has not shown benefit—versus leaving it in place for potential future use—has not been systematically addressed. Fifth, post-trial access: patients who do show benefit in a research context should have a clear plan for continued stimulation and device maintenance after trial completion. Sixth, the possibility of restoring awareness without communication or functional independence raises the question of how to assess and honor the patient’s subjective experience; caregiver-reported quality of life, while valuable, should not be considered a substitute for the patient’s own subjective experience, and efforts should be made to develop assessment tools that can detect signs of distress or wellbeing in non-communicative patients. This tension underscores why several authors in this review explicitly argue that outcome measures for future DBS/SCS trials should extend beyond the CRS-R to include family- and surrogate-reported quality-of-life and psychological wellbeing measures, and that interdisciplinary ethics oversight—rather than efficacy data alone—should guide case-by-case decisions to proceed with surgery, especially outside the context of formal clinical trials (Vanhoecke and Hariz, 2017).

It is important to recognize genuine strengths within this literature alongside its limitations. The methodological trajectory over the reviewed decade shows clear maturation: from uncontrolled single-case reports in the 2010s toward multicenter IPD meta-analyses, PRISMA-adherent systematic reviews, and mechanistically grounded neuroimaging/electrophysiology studies by the 2020s (Bergeron et al., 2025; Lee et al., 2026; Dutta et al., 2025). Several groups have also been commendably transparent about negative or null findings—for example, explicitly reporting that short-duration test stimulation fails to produce measurable behavioral change even when it modulates electrophysiology (Aihemaitiniyazi et al., 2026; Wang et al., 2023)—and about the insufficiency of current evidence to justify altering the natural history narrative given to families (Bergeron et al., 2025). Nonetheless, the absence to date of any randomized, sham-controlled trial for either DBS or SCS in DOC remains the single most important gap in this field, repeatedly acknowledged by the authors of the primary studies themselves (Yang et al., 2023; Yang et al., 2022; Xia et al., 2018), and is essential given the demonstrated capacity of this population for meaningful and sometimes delayed spontaneous recovery.

## 7 Future directions

Building on the evidence and gaps summarized above, several converging directions appear well-positioned to advance the field over the coming decade. First, adequately powered, multicenter, ideally randomized (e.g., stepped-wedge, sham-controlled, or crossover-with-blinded-assessment) trials are needed for both DBS and SCS, incorporating pre-registered stimulation protocols and standardized, etiology-stratified enrollment criteria (Liao and Gao, 2026). Second, network-based and electrophysiological patient-selection frameworks—such as the striatal-preservation and ventral-tegmental-tract engagement model for DBS (Warren et al., 2025) and central-thalamic theta-rhythm signatures that identify recovery-prone subgroups even among patients with poor initial clinical presentation (Zhang et al., 2025)—should be prospectively validated as enrollment or stratification criteria in future trials, rather than used solely for *post hoc* explanation. Third, technical innovations that reduce targeting error and improve access, including machine-learning-assisted stereotactic targeting (Singh et al., 2026) and robotic non-rigid registration for anatomically distorted brains (Liu et al., 2026), warrant validation specifically within DOC cohorts (both were validated primarily in epilepsy or healthy populations to date) and could particularly benefit resource-limited centers. Fourth, individualized, computational-model-guided stimulus design, exemplified by the neural-field-theory framework for deriving personalized ‘healthy-like’ stimulation waveforms, represents a promising but still preclinical pathway toward truly personalized neuromodulation that should be prioritized for early-phase clinical testing (Polyakov et al., 2025).

Fifth, multimodal and concurrent biomarker recording should be expanded. Simultaneous fNIRS-EEG recording in the same patients would directly test whether hemodynamic and electrophysiological signals track a common underlying process, addressing a key gap in the current literature where the two modalities have only been studied independently. Incorporating FDG-PET or hybrid PET-MRI into pre- and post-stimulation assessments would provide direct measures of cerebral glucose metabolism that complement hemodynamic and electrophysiological readouts and could help establish whether behavioral gains are accompanied by sustained metabolic reactivation. Sixth, SCS parameter optimization should be systematized: future studies should routinely report pulse width, active contact configuration, polarity, and laterality, and should systematically investigate contact-level and differential programming strategies, building on the differential target multiplexed approach (Li et al., 2024). Finally, future trials should incorporate multimodal outcome batteries—combining CRS-R, quantitative EEG/fNIRS/fMRI biomarkers, and caregiver-reported quality-of-life measures—and explicit ethical oversight frameworks from the outset, so that efficacy, mechanism, and ethical acceptability can be evaluated jointly rather than sequentially.

## 8 Conclusion

A decade of accumulating clinical, electrophysiological, and preclinical evidence supports a biologically plausible, mesocircuit-grounded rationale for using deep brain stimulation and spinal cord stimulation to promote recovery of consciousness in carefully selected patients with chronic disorders of consciousness. Across both modalities, the most consistent clinical finding is that patients in the minimally conscious state, particularly those treated relatively early after injury and with preserved neurophysiological and structural integrity, respond more favorably than those in the vegetative state/unresponsive wakefulness syndrome, with pooled efficacy in the range of 30–40% overall and up to 63–74% in MCS subgroups, and with SCS demonstrating an appreciably lower complication rate than DBS in indirect comparison (Wu et al., 2023). The accumulated evidence most strongly favors consideration of these interventions in younger MCS patients within approximately 12 months of injury who have preserved evoked potentials and structural integrity; for VS/UWS patients, the lower expected efficacy makes the additional risk of DBS harder to justify, and SCS may be the more appropriate initial invasive option where intervention is pursued. Nevertheless, the two most rigorous individual patient data meta-analyses available for DBS agree that current evidence does not establish that either modality reliably alters the natural history of neurological recovery, and no adequately powered randomized controlled trial exists for either treatment (Bergeron et al., 2025; Lee et al., 2026). The divergent etiology findings between the DBS and SCS literatures remain unresolved and should not be over-interpreted given small subgroup sizes. Continued progress will depend on multicenter randomized trials, biomarker-guided patient selection, technical refinement of targeting and stimulation parameters (including systematic reporting of pulse width and contact configuration), concurrent multimodal biomarker recording (including fNIRS-EEG and FDG-PET), and sustained attention to the distinctive ethical challenges of intervening in patients who cannot themselves consent to, or report the subjective consequences of, a partial return of awareness (Vanhoecke and Hariz, 2017).
